# Exosomal non-coding RNAs in glioma progression: insights into tumor microenvironment dynamics and therapeutic implications

**DOI:** 10.3389/fcell.2023.1275755

**Published:** 2023-11-01

**Authors:** Davide Marangon, Davide Lecca

**Affiliations:** Laboratory of Molecular and Cellular Pharmacology of Purinergic Transmission, Department of Pharmaceutical Sciences, Università degli Studi di Milano, Milan, Italy

**Keywords:** microRNA, lncRNA, circRNA, extracellular vesicles, glioma, TME, tumor-associated macrophages and microglia, glial cells

## Abstract

Gliomas are the most common and deadly types of brain tumors, known for their extensive genetic and epigenetic variability, which poses considerable challenges for pharmacological treatment. Glioma heterogeneity is also related to their intricate and dynamic tumor microenvironment (TME), which comprises a diverse array of cell types, including immune cells, vascular cells, glial cells, and neural precursors, collectively influencing tumor behavior and progression. A pivotal aspect of this intercellular communication relies on the exchange of extracellular vesicles (EVs), which contain and transfer complex molecular cargoes typical of their cells of origin, such as proteins, lipids, carbohydrates, metabolites, and non-coding RNAs (ncRNAs), that encompass microRNAs (miRNAs), long non-coding RNAs (lncRNAs), and circular RNAs (circRNAs). Glioma cells actively release EVs loaded with specific ncRNAs that can target genes and other ncRNAs in recipient cells residing within the TME. Among these recipient cells, prominent players include tumor-associated macrophages and microglia (TAMs), non-neoplastic astrocytes and endothelial cells. The intricate interplay between EVs derived from glioma cells and these recipient cells significantly contributes to the establishment of a tumor-permissive microenvironment, promoting tumor cell proliferation, migration, angiogenesis, and invasion, by targeting various downstream pathways. This review critically examines the current understanding of the intricate interplay between glioma, exosomal ncRNAs, and various components of the glioma TME. By shedding light on the roles of ncRNAs in mediating intercellular communication, this review underscores their significance in orchestrating TME transformation and highlights their potential as novel therapeutic targets for effectively tackling glioma progression.

## 1 Introduction

Gliomas are the most prevalent primary tumors of the CNS originating from glial cells, diagnosed and classified based on histopathology. Among them, glioblastoma multiforme (GBM) is the most common and aggressive primary malignant brain tumor (Taylor et al., 2019). In gliomas, cancerous cells continuously communicate with cells of the tumor microenvironment (TME), which comprises peripheral immune cells (macrophages and lymphocytes), vascular cells, glial cells, and neural precursors (NPCs), thus profoundly altering their physiological behavior. Tumor-associated macrophages and microglia (TAMs) that contribute up to 30%–40% of a brain tumor mass, are immune cells “reprogrammed” by the tumor, that release anti-inflammatory factors, promote angiogenesis and degradation of extracellular matrix, thus sustaining tumor progression, recurrence, and inhibiting response to therapy (Parmigiani et al., 2021). Non-neoplastic astrocytes can be converted into a reactive phenotype able to secrete several immunosuppressive factors, which influences tumor migration and growth (John Lin et al., 2017). In addition, once activated, astrocytes and microglia release cytokines that can boost each other’s activation. A recent analysis highlighted that alterations in oligodendrocytes and oligodendrocyte progenitor cells (OPCs) also represent an important feature of the TME. For example, in the proneural GBM subtype, characterized by high PDGFRα gene expression and frequent IDH1 mutation (Zhang et al., 2020), infiltrating oligodendrocytes that express high levels of PDGFα fuel tumor proliferation (Caruso et al., 2020). Lymphocytes, in particular T cells, play an important role in the antitumor immune response. Indeed, increased infiltration of T cells is associated with prolonged survival of GBM patients. However, factors from the TME drive T cell exhaustion and dysfunctional metabolic states (Wang et al., 2021), leading to the prevalence of immunosuppressive phenotypes (T-helper 2 and T-regs). Tumor vasculature nourishes glioma as well and provides a specialized niche for glioma cells, that, in response to environmental signals, become self-renewing glioma stem cells (GSCs), representing the key actors in glioma expansion and radioresistance (Ong et al., 2017). Differently from the other cells of the TME, NPCs are able to delay tumor growth by triggering cell death (Glass et al., 2005).

**FIGURE 1:**
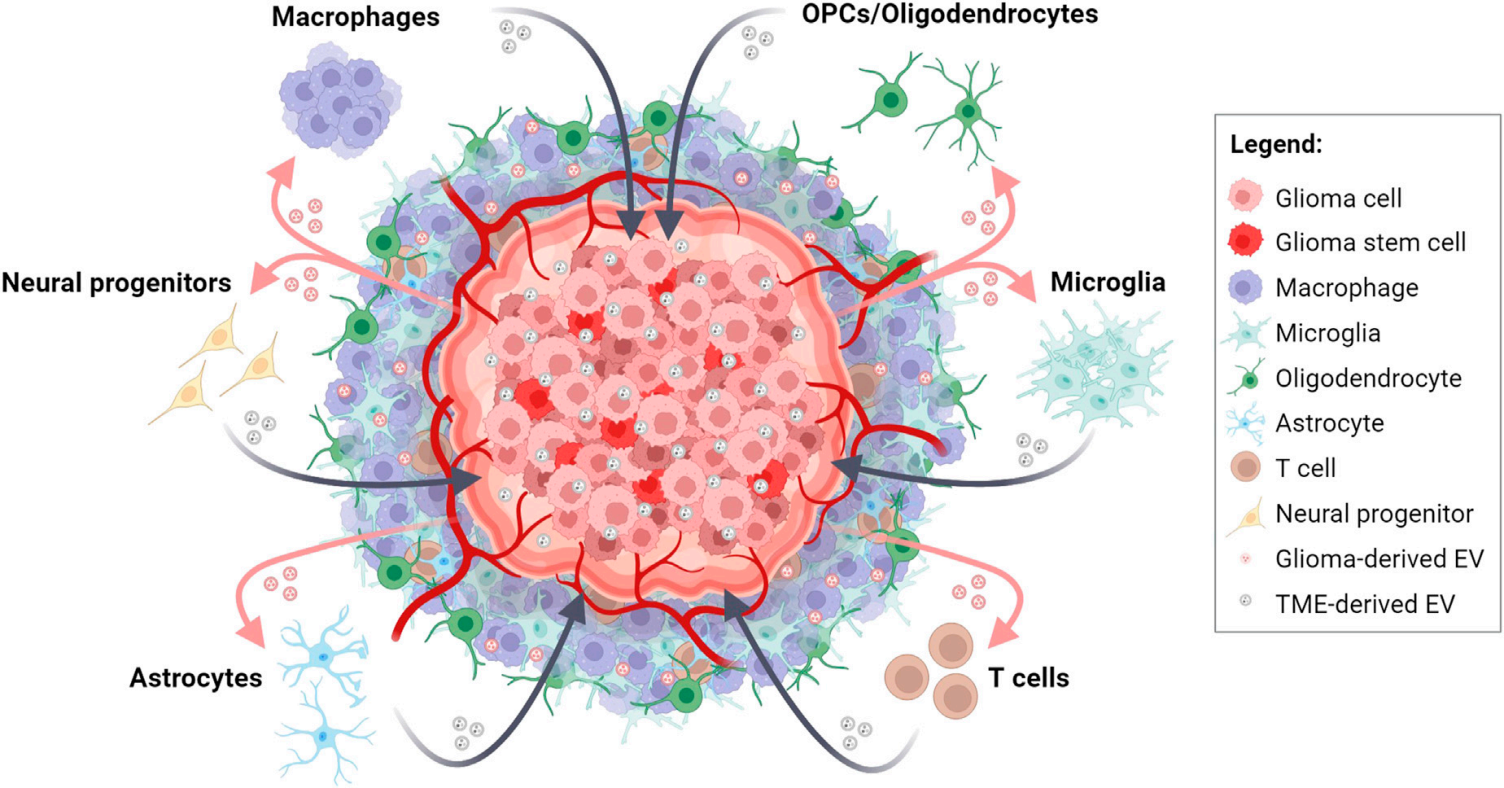
Bidirectional EV-based communications in glioma and tumor microenvironment (TME) crosstalk. Schematic representation depicting the intricate exchange of molecular information between glioma cells and cells of the TME through the release and uptake of EVs. This dynamic interplay contributes to tumor progression, TME transformation, immune response modulation, and therapeutic resistance in glioma. Created with BioRender.com.

Glioma and TME cells use different communication routes that facilitate tumor progression, including receptor-ligand interactions, release of soluble factors, such as cytokines, chemokines, and metabolites, and exchange of extracellular vesicles (EVs) (Sharma et al., 2023). Recently, EVs have drawn much attention due to their ability to carry various bioactive molecules not only in the tumor environs but also at distant sites (Figure 1), altering expression of tumor promoting and tumor suppressing genes in recipient cells (Chulpanova et al., 2022). EVs are lipid bound vesicles released by most cell types that, based on their biogenesis, can be broadly categorized in ectosomes (100–1,000 nm), exosomes (30–120 nm) and apoptotic bodies (50–5,000 nm) (Marangon et al., 2022; Jeppesen et al., 2023). Ectosomes originate from direct plasma membrane budding; their release requires the separation from the plasma membrane and mainly depends on the interaction between actin and myosin and adenosine triphosphate (ATP)-mediated energy supply. Instead, exosomes biogenesis starts from the formation of intraluminal vesicles in late endosomes/multivesicular bodies (MVBs). Then, MVBs fuse either with lysosomes to be degraded or with the plasma membrane to release exosomes into extracellular spaces. Upon reaching recipient cells, EVs can either fuse with the plasma membrane, or be internalized by target cells via endocytosis, phagocytosis, micropinocytosis, or lipid-raft-mediated endocytosis [for review, see: Gurung et al. (2021) and Liu and Wang (2023)]. EVs mediate the cell communication by transfering their content, which comprises a specific set of proteins, lipids, and nucleic acids, most of which non-coding RNAs (ncRNAs) (Huo et al., 2021), to recipient cells. NcRNAs, such as microRNAs, long non-coding RNAs (lncRNAs), and circular RNAs (circRNAs), are post-transcriptional regulators implied in a wide range of physiological and pathological processes, including glioma development (Rajabi et al., 2022). miRNAs are small RNA molecules that bind to the 3′UTR of their target transcripts, resulting in mRNA degradation or translational repression (Lecca et al., 2016; Marangon et al., 2021). LncRNAs, initially considered as “junk RNA,” can interact with DNA, RNA, and proteins, modulating chromatin structure and transcription, affecting RNA splicing, stability, and translation (Statello et al., 2021). CircRNAs are a class of covalently closed circular ncRNAs involved in complex biological processes, including regulation of pre-mRNA splicing, RNA binding protein sequestration, and IRES-mediated CAP-independent translation (Misir et al., 2022). Interestingly, both circRNAs and lncRNAs can bind miRNAs as competitive endogenous RNAs (ceRNAs) (Ma et al., 2023), leading to the generation of a complex network consisting of multiple circRNA/lncRNA-miRNA-mRNA axes, which could ultimately regulate hundreds of genes. During EV biogenesis, these multiple RNA species are packaged into different subclasses of EVs. Cytoplasmic localization, small size, high abundance, affinity for membranes and RNA-binding proteins (RBPs) favor the incorporation of a given RNA molecule into EVs. Of note, in mammalian cells, there are more than 500 RBPs, which represent about 25% of the total protein content of EVs (O'Brien et al., 2020). Different RBPs exhibit binding preferences for different RNA sequence motifs, but a full understanding of these binding interactions still has to be established (Fabbiano et al., 2020). Other sorting signals include RNA and/or RBP modifications, such as ubiquitylation, sumoylation, phosphorylation and uridylation. A growing number of studies have identified additional proteins involved in EV loading, such as KRAS, HuR, MEX3C, Ago2, and IGF2BP1 (Fabbiano et al., 2020).

Recently, it has been shown that ncRNAs encapsulated in glioma-derived EVs (GDEVs) can drive mutations and epigenetic modifications in various pathological phases of glioma progression (Cheng et al., 2020) and in the transformation of the cells composing the TME. On the other hand, exosomal transfer of ncRNAs from both TAMs and reactive astrocytes to glioma cells can initially counteract tumor progression, but then it promotes tumor malignancy. In this review, we will describe the most recent evidence about the role of exosomal ncRNAs and their implications for tumorigenesis, and we will highlight the potential relevance of this communication in the pharmacological treatment for glioma.

## 2 Exosomal ncRNAs from glioma cells sustain glioma progression

The first level of communication takes place between cancer cells. In the T98G glioblastoma cell line, miR-148a, a miRNA found upregulated in serum exosomes from GBM patients compared to healthy volunteers (Cai et al., 2018), was described as an oncomiR, involved in cell proliferation, migration, and epithelial–mesenchymal transition (EMT). The pro-tumorigenic effects of miR-148a were shown to be mediated by its interaction with target transcripts encoding for CADM1, a protein involved in cell adhesion, and DLGAP1, a scaffolding protein involved in microtubule dynamics (Li Y. et al., 2019). These data suggest that GBM cells can utilize exosomes to promote cancer cell proliferation and metastasis through miR-148a.

The lncRNA LINC00470, overexpressed in several tumors, including gliomas (Wu et al., 2020), was pathologically overrepresented in GDEVs and was associated with disease severity and postoperative survival time of glioma patients. Infusion of GBM-derived exosomes worsened tumor progression in nude mice, inhibiting autophagy; this effect was rescued by silencing LINC00470. The proposed mechanism is the competitive binding of LINC00470 to miR-580-3p, a negative regulator of the WEE1 gene and of the PI3K/AKT/mTOR pathway, that enhances GBM proliferation (Ma et al., 2021). A similar “sponging” mechanism was described for ROR1-AS1, a lncRNA whose overexpression in glioma tissues was associated to poor prognosis (Chai et al., 2020). ROR1-AS1 was also identified in exosomes derived from glioma cells *in vitro*. Functional analysis showed that exosomal ROR1-AS1 promoted the progression of glioma cell lines SHG44 and U251, acting as a sponge of miR-4686, a potential tumor-suppressor, and inhibiting its activity. Tumorigenesis experiments *in vivo* have also confirmed that exosomes containing ROR1-AS1 promoted glioma development by blocking the miR-4686 axis.

Exosome-derived circ-0001445 was shown to be taken up by glioma cells and to act as a sponge for miR-127-5p, thus upregulating the expression of sorting linker protein 5 (SNX5), which promotes glioma migration and invasion (Han et al., 2021). Similarly, circZNF652 is a circular RNA upregulated in human GBM tissues and abundantly stored in exosomes (Liu et al., 2022). Patients with high expression of circZNF652 showed worse prognosis and higher invasiveness. *In vitro* and *in vivo* analyses demonstrated that its action is mediated by a sponging effect on miR-486-5p that leads to abnormally high levels of SERPINE1, a fibrinolytic serine protease inhibitor associated with several malignancies (Liu et al., 2022).

High cellular heterogeneity due to continuous mutations inside the tumor stroma provides high adaptation to standard treatments over time, and highly aggressive recurrences located close the original lesion take place in almost all the patients (Buruiana et al., 2020). For this reason, the current clinical protocols for glioblastoma treatments, that require surgical resection followed by chemotherapy and radiotherapy, enhance the survival of only a few months (Stupp et al., 2005). The primary actors in radio- and chemo-resistance are GSCs, a small subpopulation of tumor cells within gliomas that exhibit stem cell–like properties. In a recent study, GSC-EVs derived from three different glioma patients were found to significantly enhance the radiation resistance of non-GSC glioma cells. GSC-EV characterization identified 25 highly expressed miRNAs in all three GSC lines and 8 of them (miR-320e, miR-520f-3p, miR-363-3p, miR-144-4p, miR-16-5p, miR-495-3p, miR-23a-3p, miR-155-5p) showed a seed sequence able to target the onco-suppressor PTEN, even if their role in the etiopathogenesis has not been fully evaluated (Ma et al., 2022). Another recent study identified four miRNAs (miR-1280, miR-1238, miR-938, and miR-423-5p) overexpressed in temozolomide (TMZ)-chemoresistant compared to TMZ-chemosensitive tissues. Among them, miR-1238 was found dramatically upregulated in recurrent GBM samples compared to primary GBM tissues, and in exosomes of TMZ-sensitive compared to resistant cells, where it correlated to TMZ resistance (Yin et al., 2019). Further molecular studies showed that exosomal transfer of miR-1238 from resistant to sensitive cells could confer chemoresistance by activating the EGFR-PI3K-AKT-mTOR pathway. Pharmacological strategies aimed at neutralizing the tumorigenic effects of these miRNAs could open new perspectives in advanced glioma therapy.

Accumulating evidence indicates that miRNA sponging by exosomal circRNAs and lncRNAs is a very common mechanism enhancing chemoresistance to TMZ in glioblastoma. Exosomes isolated from various TMZ-resistant GBM cells were shown to be enriched in circCABIN1 (Liu et al., 2023), circWDR62a (Geng et al., 2022), SBF2-AS1 (Zhang et al., 2019), and circ-METRN (Wang X. et al., 2021), which act respectively as ceRNA for miR-637, miR-370-3p, miR-151a-3p, and miR-4709-3p. MiRNA sponging leads to the disinhibition of their endogenous targets (OLFML3, MGMT, XRCC4, GRB14, respectively), which are involved in stemness reprogramming, proliferation and DNA double-strand break repair in cancer cells, thus contributing to spread TMZ resistance to chemoresponsive GBM cells.

Beyond their role in resistance to therapy, GSC-derived exosomal ncRNAs are able to trigger several pro-tumor processes, such as growth, invasion and angiogenesis. Recent studies have shown that GSC-derived exosomes are enriched in miR-155-5p and miR-26a, and that they can be horizontally transferred to surrounding glioma and brain microvascular endothelial cells (BMECs). miR-155-5p transfer to glioma cells markedly reduced the expression of acetyl-CoA thioesterase 12 (ACOT12), a tumor suppressor gene, and contributed to the promotion of mesenchymal transition (Bao et al., 2022). In BMECs, miR-26a overexpression contributed to enhanced proliferation, migration and tube formation by targeting PTEN (Wang et al., 2019).

Hypoxia, a typical feature of GBM, is known to deeply influence glioma progression malignancy, and resistance to therapy, and to alter the production and composition of GDEVs. A recent study demonstrated that hypoxic (H)-GDEVs promoted normoxic (N) glioma migration and invasion both *in vitro* and *in vivo* (Qian et al., 2022). This effect was partly mediated by miR-1246 and miR-10b-5p, enriched in H-GDEVs and whose expression in glioma patients was already correlated to poorer prognosis (Nix et al., 2021; Wang et al., 2021), by directly targeting FRK and TFAP2A, respectively. Interestingly, further studies have shown that hypoxia promoted the enrichment of miR-1246 in GDEVs by increasing its transcription and selective exosomal sorting through upregulation of two HIF-1α-dependent factors, namely, the POU class 5 homeobox 1 (POU5F1) and the heterogeneous nuclear ribonucleoprotein A1 (hnRNPA1) (Qiu et al., 2021). Another recent study has shown that exosomal miR-106a-5p derived from hypoxia glioma cells could reduce the sensitivity of glioma cells to TMZ through PTEN downregulation (Wu et al., 2022).

**FIGURE 2:**
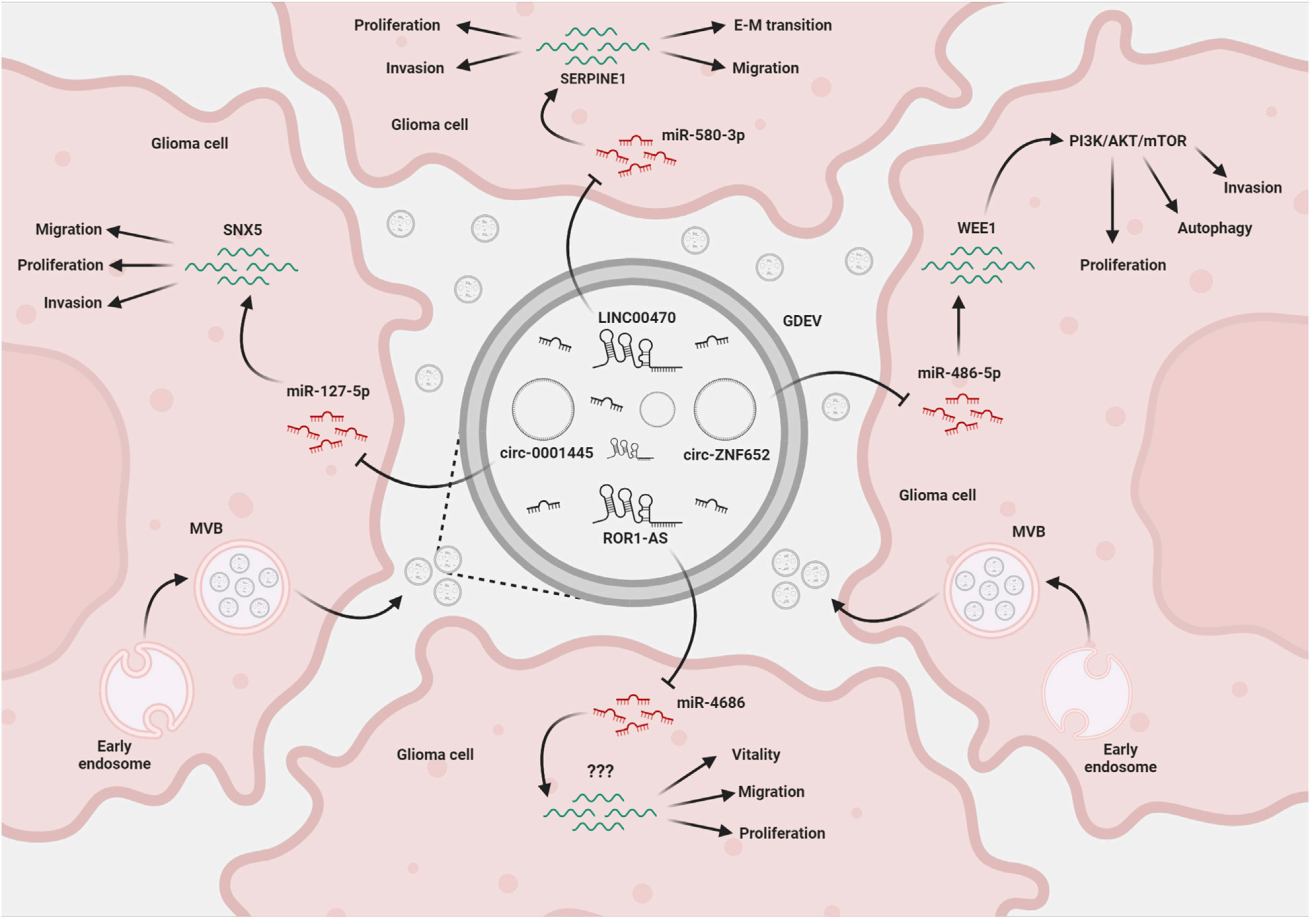
EV-mediated exchange of ncRNAs in glioma. Glioma cells release EVs (GDEVs) containing miRNAs, that target transcripts and downstream pathways in other glioma cells, affecting vital processes such as migration, invasion, proliferation, autophagy, and vitality. Long non-coding RNAs (lncRNAs) and circular RNAs (circRNAs) within the EV cargo act as intracellular microRNA sponges, forming a complex regulatory network that affects glioma growth. The figure represents some of the mechanisms described in the text. MVB, multivesicular bodies; EMT, epithelial-mesenchymal transition; Created with BioRender.com.

Recent studies have shown that hypoxic conditions are able to influence exosomal abundance of specific lncRNAs and circRNAs. LncRNA sequencing analysis of exosomes derived from H-GSC and N-GSC identified the long intergenic nonprotein-coding RNA 1060 (Linc01060) as one of the most upregulated lncRNA (Li J. et al., 2021). Linc01060 expression was found inversely related to patient prognosis and, when transferred through exosomes, accelerated proliferation, migration, and invasion of tumor cells both *in vitro* and *in vivo*. Mechanistically, Linc01060 directly binds the myeloid zinc finger 1 (MZF1), preventing its ubiquitination-mediated degradation, accelerating its nuclear translocation, and promoting MZF1-mediated c-Myc transcriptional activities. Very recently, a circRNA microarray analysis allowed to identify circ101491 as one of the most expressed circRNAs in hypoxic GSC-secreted exosome (Zhang et al., 2023). Overexpression of circ101491 significantly increased, while its downregulation reduced viability, invasion, and migration of glioma cells. Further experiments revealed that circ101491 might upregulate oncogene EDN1 expression by sponging miR-125b-5p. In summary, glioma cell communication involves complex interactions driven by exosomal ncRNAs, which impacts on various aspects of tumor progression, making them potential therapeutic targets. A schematic representation of these interactions has been reported in Figure 2.

## 3 Glioma cells transform the TME through exosomal ncRNAs

Among the many cell types found in TME, TAMs play a significant role in glioma progression. Despite some studies suggested that microglia/macrophages have the potential to counteract glioma development, evidence have shown that their immune functions become pro-tumorigenic. Thus, after interacting with tumor cells, TAMs cannot engage an efficient immune response, and show M2-like phenotypic activation changes with increase in the release of anti-inflammatory cytokines, such as IL-10 and TGF–β, arginase-1 (Arg-1), and matrix metalloproteinases, thus supporting growth and invasion associated with tumor progression (Feng et al., 2022).

Both *in vitro* and *in vivo* experiments revealed that exosomes released by glioma cells can be avidly taken up by other cells and contribute to the malignant alteration of the TME. GDEVs carry high levels of miR-21, a crucial oncomiR that promotes invasion and cell survival in GBM cells, through the downregulation of the tumor suppressor gene IGFBP3 and the enhancement of metalloprotease activity (Yang et al., 2014). *In vitro* studies have shown that exposure of microglia/macrophages to GDEVs elevated the levels of miR-21 and decreased levels of a common mRNA target encoding c-Myc (van der Vos et al., 2016). *In vivo* experiments in miR-21-null mice, have shown that early after injection, resident microglia and macrophages efficiently internalized the vesicles and downregulated specific miR-21 target transcripts associated to proliferation (Abels et al., 2019). Exosomal miR-21 derived from GSCs could also promote the angiogenic ability of endothelial cells by stimulating the VEGF signalling pathway (Sun et al., 2017). Similarly, miR-148a-3p and miR-182-5p were found to be enriched in GDEVs and transferred to human umbilical vein endothelial cells (HUVECs), where they downregulate the expression of ERRFI1, KLF2 and 4, thus activating multiple signaling pathways promoting cell proliferation and angiogenesis (Li et al., 2020; Wang et al., 2020). Interestingly, GBM cells can modulate microglia activity also in response to radiotherapy. TME can be aberrantly altered by the exosomal transfer of circ_001381 from GBM cells to microglia and the activation of a circ_001381/miR340-5p/Arg1 axis (Zhang et al., 2022). These changes induce microglia to acquire a pro-tumoral phenotype, characterized by an increased expression of inflammatory mediators and a reduction of the phagocytic capability.

Recently, whole-transcriptome sequencing of extracellular vesicles derived from CSF of glioma patients performed in parallel to matched glioma tissues revealed that some high-abundant miRNAs, such as miR-1298-5p, miR-122-5p, miR-204-5p and miR-3184-3p (Xu et al., 2022), were almost exclusively secreted in exosomes, whereas some high-abundant tumor-promoting miRNAs, such as miR-9-5p, were mainly retained in tumor cells (Qi et al., 2022). miR-1298-5p and miR-3591-3p, enriched in CSF exosomes, were able to suppress glioma progression *in vitro* and vivo (Li et al., 2022; Qi et al., 2022), but also to exert an immunosuppressive activity (i.e., polarize toward the M2-like phenotype) on both macrophages and myeloid-derived suppressor cells (MDSCs), a population of immature myeloid cells that suppress the function of T cells. Conversely, miR-9-5p, retained in glioma cells, promoted migration, helped progression of tumor cells, and induced M1 polarization in transfected macrophages (Qi et al., 2022). Other miRNAs, such as miR-3184-3p and miR-1246, were found enriched in exosomes and upregulated in CSF and glioma tissues (Qiu et al., 2021; Xu et al., 2022). On the one hand, they can directly promote proliferation, migration, and invasion by inhibiting apoptosis in glioma cells. On the other hand, when transferred to macrophages, they can induce an M2-like phenotype, which further aggravates tumour progression. These examples suggests that exosomal release can have a double function; on one hand it sustains glioma progression creating the most suitable microenvironment instructing the surrounding cells not to hamper tumor proliferation, on the other hand it removes tumor suppressive elements from tumor cells, thus facilitating their invasiveness.

As mentioned above, hypoxia contributes to alter exosome production and composition. Of note, hypoxia-induced exosomal ncRNAs profoundly affect not only the behaviour of tumor cells, but also that of other cells in the TME. It has been shown that, compared to N-GDEVs, H-GDEVs contain higher levels of both IL-6 and miR-155-3p, and induce M2-like macrophage polarization via the IL-6-pSTAT3-miR-155-3p-autophagy-pSTAT3 positive feedback loop, promoting glioma progression (Xu et al., 2021). Another study identified 20 hypoxia-induced exosomal miRNAs. Of these, miR-10a, miR-21, miR-29a and miR-92a were able to induce myeloid cell differentiation and expansion by targeting the Rora/IκBα/NF-κB, Pten/PI3K/AKT, Hbp1/cell cycle and Prkar1a/PKA/p-STAT3 pathways, respectively, promoting their immunosuppressive functions in glioma-bearing mice (Guo et al., 2018; Guo et al., 2019). Of note, their inhibition in glioma cells attenuated myeloid cell expansion and differentiation in spleen and tumor tissues *in vivo*.

Although many lncRNAs were described overexpressed in GBM tumors, only a few of them were reported to be trafficked into GDEVs and to contribute to spread tumor to the surrounding cells. The long intergenic ncRNA CCAT2 (linc-CCAT2), overexpressed in glioma tissues, was demonstrated to contribute to promote angiogenesis via exosomal communication. U87-derived exosomes, enriched in linc-CCAT2, were shown to induce HUVEC migration, proliferation, tubular-like structure formation *in vitro* and arteriole formation *in vivo* through the activation of VEGFA and TGFβ, and inhibition of antiapoptotic players, such as Bax and caspase-3 (Lang et al., 2017). SNHG15, a lncRNA upregulated in GBM associated to poor prognosis and TMZ-resistance has been demonstrated to actively contribute to angiogenesis and immune inhibition in the TME (Li Z. et al., 2019). *In vitro* studies highlighted that SNHG15 silencing in GBM cells was able to significantly increase their sensitivity to TMZ treatment and to reduce their ability to promote M2-phenotype in microglial cells, raising the hypothesis that SNHG15 may be transferred to microglia through EVs or act as a ceRNA for exosomal miRNAs. Indeed, its tumorigenicity is mediated by the direct inhibition with miR-627-5p, a tumor suppressor that inhibits the cyclin dependent kinase CDK6. The pharmacological intervention in this SNHG15/miR-627-5p/CDK6 axis has been proposed for the treatment of TMZ-resistant GBM (Li Z. et al., 2019). Beyond their miRNA sponging activity, lncRNAs may also interact with RNA binding proteins and activate disease-relevant pathways. In this context, a recent study elucidated the role of the TMZ-associated lncRNA (lnc-TALC) in the promotion of GBM resistance to TMZ (Li Z. et al., 2021). Lnc-TALC can be incorporated into GDEVs and transmitted to TAMs, to promote immunosuppressive phenotype of microglial cells. This effect was found related to its interaction with ENO1, to the phosphorylation of p38 MAPK and to the secretion of the complement components C5/C5a, which promoted the repair of TMZ-induced DNA damage, leading to chemotherapy resistance. These studies suggest that combination therapy strategies targeting different lncRNAs might help to overcome TMZ resistance in GBM.

GDEVs are also able to activate surrounding astrocytes. EVs purified from two distinct human glioma cell lines were found enriched in the lncRNA activated by TGF-β (lncRNA-ATB). When incubated with NHA cells, a type of normal human brain astrocytes, GDEVs were efficiently delivered and lncRNA-ATB activated astrocytes through the suppression of miR-204-3p, thus stimulating the secretion of TGF-β and glioma cell migration and invasion (Bian et al., 2019).

Although T cells play an important role in the development and prognosis of GBM, the effects of GDEVs (and their RNA cargo) on the different subtypes of T cells are poorly known. In unfractionated peripheral blood mononuclear cells (PBMCs), GDEVs can inhibit T cell activation, proliferation and Th1 cytokine production and enhance proliferation of purified CD4^+^ T cells (Domenis et al., 2017). Importantly, the removal of MDSCs cell fraction from PBMCs restored T cell proliferation. In addition, it was recently shown that T cells do not internalize tumor-derived exosomes; consequently, exosomes could deliver signals to receptors on the cell surface, ultimately resulting in alterations of the mRNA profile (Muller et al., 2016).

**FIGURE 3:**
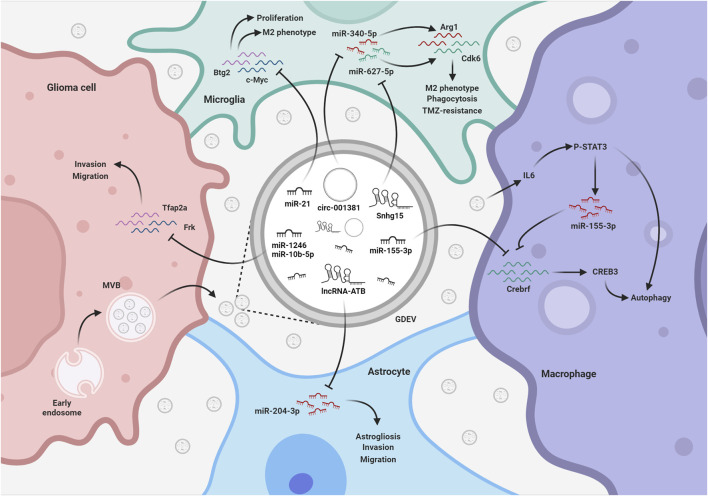
EV-mediated exchange of ncRNAs among glioma and TME. GDEVs transfer ncRNAs also to microglia, macrophages, and astrocytes within the TME. These ncRNAs promote astrogliosis and the development of a tumor-supportive microglia phenotype, reducing phagocytosis and autophagy, thereby shaping a favorable environment for glioma progression. The figure represents some of the mechanisms described in the text. MVB, multivesicular bodies; TMZ, Temozolomide; Created with BioRender.com.

As summarized in Table 1 and represented in Figure 3, these data have shown that glioma cells use EVs and ncRNA cargoes to change the reactivity of potentially harmful microglia, macrophages, and astrocytes, to efficiently expand in the surrounding area. Considering the key role of EV-based communication in glioma progression, a potential therapeutic strategy could be based on the suppression of such communication. Promising results have been obtained in cancer cell lines and xenograft models modulating the exosome release pathway (Kosaka et al., 2013; Messenger et al., 2018).

**TABLE 1:** Summary of glioma EV-derived ncRNAs, their targets and effects on cells of the TME.

Source	Recipient cells	Neuronal ncRNAs	ncRNA targets	Mechanism of action	References
Primary GBM cells	Primary mouse microglia	miR-21	c-Myc, Btg2, Igfbp3	Promotion of tumor supportive phenotype	Yang et al. (2014); van der Vos et al. (2016); Abels et al. (2019)
U251 GBM and U87 cell lines	HUVECs	miR-148a-3p and miR-182-5p	Errfi1, Klf2, Klf4	Promotion of cell proliferation and angiogenesis	Li et al. (2020); Wang et al. (2020)
U251 and U87 GBM cell lines	human HMC3 microglia cell line	circ_001381	miR-340-5p/Arg1	Reduction of the phagocytic capability	Zhang et al. (2022)
U251 and U87 GBM cell lines	human U937 and THP-1 monocyte cell lines	miR-155-3p	Crebrf	M2-like macrophage polarization via STAT3 activation	Xu et al. (2021)
U87 GBM cell lines (hypoxic)	Mouse bone marrow and MDSC cells	miR-10a, miR-21, miR-29a and miR-92a	Rora, Pten, Hbp1, Prkar1a	Induction of myeloid cell differentiation, expansion and immunosuppressive functions	Guo et al. (2018); Guo et al. (2019)
U87 GBM cell line	HUVECs	linc-CCAT2	Unknown	Activation of VEGFA and TGFβ, and inhibition of antiapoptotic players	Lang et al. (2017)
Primary GBM cells	Human HMC3 microglia cell line	SNHG15	miR-627-5p/Cdk6	Decrease sensitivity to TMZ treatment and promotion of M2-phenotype	Li et al. (2019b)
Human LN229 and mouse GL261 cell lines	Human HMC3 microglia cell line	lnc-TALC	ENO1	The promotion of GBM resistance to TMZ	Li et al. (2021c)
A172 and U87 GBM cell lines	Primary human astrocytes	lncRNA-ATB	miR-204-3p	Promotion of astrocyte activation	Bian et al. (2019)

## 4 Janus-faced role of TME-derived ncRNAs in glioma progression

The communication between glioma and TME is bidirectional. As soon as the resident immune cells identify tumor cells, they contact them, activate pro-apoptotic pathways, release pro-inflammatory factors and EVs. Recent studies have shown that EVs released by healthy astrocytes are able to reduce glioma progression. A first study showed that miR-152-3p and miR-143-3p propagate from normal astrocytes to C6 glioma in a connexin 43-dependent and -independent manners, respectively (Fukuda et al., 2021). Although the expression of these miRNAs did not alter glioma cell proliferation, a significant reduction of cell migration and invasion was observed. Similarly, Serpe and colleagues showed that treatment of GL261 glioma cells with astrocyte-derived EVs (ADEVs) significantly reduced cell viability. In contrast, EVs isolated from astrocytes treated with the glioma-conditioned medium (GCM) increased GL261 cell viability, indicating that only EVs released by healthy astrocytes exert an antitumoral effect (Serpe et al., 2022). Likewise, treatment of glioma-bearing mice with ADEVs reduced, while treatment with GCM-ADEVs increased, tumor volume, tumor cell proliferation and invasion. One of the mechanisms responsible for these effects is the transfer of miR-124, a miRNA with a known tumor suppressor role, abundantly expressed in the CNS, and highly enriched in microglia-derived EVs. In this context, miR-124 reduced the expression of VRAC, one of the multiple regulators of cell shape and migration, and ultimately hindered glioma growth (Serpe et al., 2022).

Despite these data agree with the concept that astrocytes have the potential to acutely react to brain tumor promoting the restoration of brain functionality, several studies have documented that, after some time, astrocytes become the target of tumor-released factors, which modify them towards a pro-tumoral program (Henrik Heiland et al., 2019; Mega et al., 2020) and promote the release of EVs supporting tumor growth. In line with this, it has been shown that *in vivo* ADEVs mediate intercellular transfer of PTEN-targeting miRNAs to tumor cells, leading to reduction of PTEN expression and promotion of brain metastasis (Zhang et al., 2015). Five miRNAs in the miR-17–92 cluster (miR-17, miR-19a, miR-19b, miR-20a and miR-92) were previously demonstrated to target PTEN (Liu et al., 2014). Cell specific knockdown of the miR-17–92 cluster in astrocytes blocked PTEN downregulation in brain metastatic tumor cells *in vivo* and significantly suppressed brain metastasis growth. Interestingly, PTEN levels in PTEN-loss brain metastatic tumor cells were restored after exiting the brain microenvironment, confirming that tumor cells underwent non-autonomous PTEN downregulation by astrocyte-derived PTEN-targeting miRNAs. This adaptive PTEN loss in brain metastatic tumor cells led to an increased secretion of the chemokine CCL2, which recruits IBA1-expressing myeloid cells that reciprocally enhanced the outgrowth of brain metastatic tumor cells via enhanced proliferation and reduced apoptosis (Zhang et al., 2015).

Depending on the state of the cell of origin, macrophage/microglia-derived EVs can either spread pro-inflammatory stimuli, as shown in Alzheimer’s disease and ischemia (Gabrielli et al., 2022; Marangon et al., 2022), or mediate immunosuppressive functions, as happens in GBM (Wang et al., 2022). Despite these cells have the potential to exert an antitumorigenic role, whether they actually counteract glioma growth in the very early stage of development still needs to be clearly demonstrated. A recent paper evaluated the role of M1-derived exosomal ncRNAs by performing a circRNA microarray analysis of exosomes derived from macrophages overexpressing the recombination signal binding protein-Jκ (RBP-J), a transcriptional regulator promoting classical M1 macrophage polarization activated by Notch signalling. Among the differentially expressed circRNAs, circBTG2 was found particularly enriched in exosomes and upregulated in exosomes derived from RBP-J overexpressing compared to wild type macrophages (Shi et al., 2022). Further studies showed that exosomal circBGT2 was able to suppress glioma cell proliferation and invasion via the miR-25-3p/PTEN pathway, suggesting that it may represent a new potential target for glioma therapy.

Microglia EVs uptake observed in astrocytes and glioma cells led to reduction of both astrogliosis and release of pro-tumoral factors by glioma (Serpe et al., 2021). As previously mentioned, microglia EVs are enriched in miR-124, suggesting its involvement in their anti/pro-tumorigenic effect and that modulation of miR-124 levels could represent a promising therapeutic approach to hamper glioma progression. This hypothesis has been confirmed by two different experimental approach. In the former, the authors took advantage of a miR-124 inhibitor, that specifically binds and inhibits the endogenous miR-124 activity and showed that EVs derived from anti-miR-124-transfected microglial cells failed to reduce glioma growth (Serpe et al., 2021). In the latter, the authors have produced miR-124-enriched EVs and have used them to treat a 3D microfluidic glioblastoma microenvironment composed by GBM cells, microglia and natural killer (NK) cells, showing that miR-124 EVs reduced tumor cell migration, ameliorated the profile of cytokines and chemokines secreted, and enhanced NK cell intratumoral infiltration (Hong et al., 2021).

Once reprogrammed by the TME, TAMs start to release EVs that contribute to immune suppression, thus favoring GBM immune escape (Catalano et al., 2022). In this context, a recent paper has shown that the exposure of glioma cells to M2 microglial EVs enhanced their growth, migration, and invasion capabilities, by affecting the expression of circadian genes, such as Bmal1 and Clock. Further investigations revealed that miR-7239-3p, upregulated in M2 microglial EVs, enters glioma cells and is responsible for Bmal1 gene downregulation (Li X. et al., 2021). Selective inhibition of miR-7239-3p reverted the tumor-promoting effect of M2 microglial EVs, confirming the key role of miR-7239-3p in glioma progression. EVs released by TAMs can contribute to glioma progression not only because of their enrichment in pro-tumoral effectors, but also because they lack factors that counteract glioma growth and invasion. This is the case of EVs derived from M2-polarized macrophages, which were found depleted in miR-15a and miR-92a compared to unstimulated macrophages (Yao et al., 2021). Interestingly, EVs derived from M2 macrophages transfected with miR-15a and miR-92a mimics failed to promote migration and invasion of glioma cell lines compared to EVs derived from control M2 macrophages. The same study has also shown that the anti-tumoral effects of miR-15a and miR-92a are mediated by the downregulation of their direct targets Ccnd1 and Rap1b, respectively, thereby blocking the PI3K/AKT/mTOR signaling pathway, known to promote glioma invasion and migration (Yao et al., 2021). Taken together, these results indicate that restoring the proper miRNA levels in TAMs could be considered as a promising therapeutic approach to limit glioma progression.

Proliferation and clonogenic potential of GSCs are influenced by EVs released by another stromal component of glioblastoma, namely, the glioma-associated human mesenchymal stem cells (GA-hMSCs). This was demonstrated *in vitro* on GA-hMSCs and GSCs obtained by surgical specimens, and *in vivo*, implanting GSCs pre-treated with GA-hMSC–derived exosomes in the frontal lobe of nude mice. A significant increase in tumor volume and reduction in survival were found in the group with pre-treated GSCs. Analysis of the exosomal content identified miR-1587 as the responsible of these effects on GSCs, in part through the downregulation of the tumor-suppressive nuclear receptor corepressor NCOR1 (Figueroa et al., 2017).

The crosstalk between glioma cells and oligodendrocytes has been demonstrated by the observation that tumor cells use white matter tracts to disseminate into the brain and escape radiotherapy, precluding a complete tumor eradication (Parmigiani et al., 2021). Moreover, after complete removal of the tumor mass, recurrences commonly occur in the white matter around the tumor removal cavity. At this site, called “border niche,” oligodendrocytes and OPCs tend to accumulate, promoting stemness and chemo-radioresistance of GBM cells (Hide et al., 2018). In addition to OPC recruitment, also axonal degeneration, demyelination and microglia activation were described (Brooks et al., 2021), similarly to pathogenetic events occurring in demyelinated lesions (Angelini et al., 2021). Treatment of glioma cells with OPC-derived conditioned medium significantly increased the expression of typical stemness genes (e.g., Nanog, Sox2, Oct3/4, and Bmi1), sphere-forming ability, and cell viability of glioma cells and promoted chemo-radioresistance (Hide et al., 2018). Although the role of soluble factors, such as FGF1 and EGF, has been shown, the contribution of OPC-derived EVs and their RNA content is totally unknown. Interestingly, a miRNome analysis comparing the border niche to both tumor and more distal areas showed that the miRNAs with the highest levels of expression in the border niche (i.e., miR-219-5p, miR-219-2-3p, and miR-338-3p) were related to oligodendrocyte differentiation (Hide et al., 2018), suggesting that their transfer could be involved in the supportive role of oligodendrocytes towards tumor growth.

NPCs, proposed as candidate cell-of-origin of gliomas, have also shown the ability to migrate into hypoxic areas of tumors, thus emerging as attractive cellular targets for glioma treatment. In this context, Adamus et al. (2022) tested exosomes derived from NPCs (NPDEs) as vehicles for the delivery of oligonucleotide therapeutics into the TME. In this study NPDEs were loaded with CpG-STAT3ASO, a synthetic antisense oligonucleotide that inhibit STAT3 expression. Compared to native NPDEs, the CpG-STAT3ASO-loaded exosomes potently stimulated immune activity of dendritic cells and macrophages, promoting NF-κB signaling and interleukin-12 production, and inhibited subcutaneous tumor growth more effectively than the equivalent amount of oligonucleotide alone. NPDEs have also been utilized to deliver miR-124-3p to glioma cells *in vitro* and *in vivo* (Qian et al., 2022). NPDE loaded with miR-124-3p significantly inhibited glioma cell proliferation, invasion and migration, by inhibiting the Flot2/Akt pathway. These results indicate that NPCs and NPDEs could represent a clinically relevant strategy to improve delivery and safety of RNA therapeutics for glioma treatment.

**FIGURE 4:**
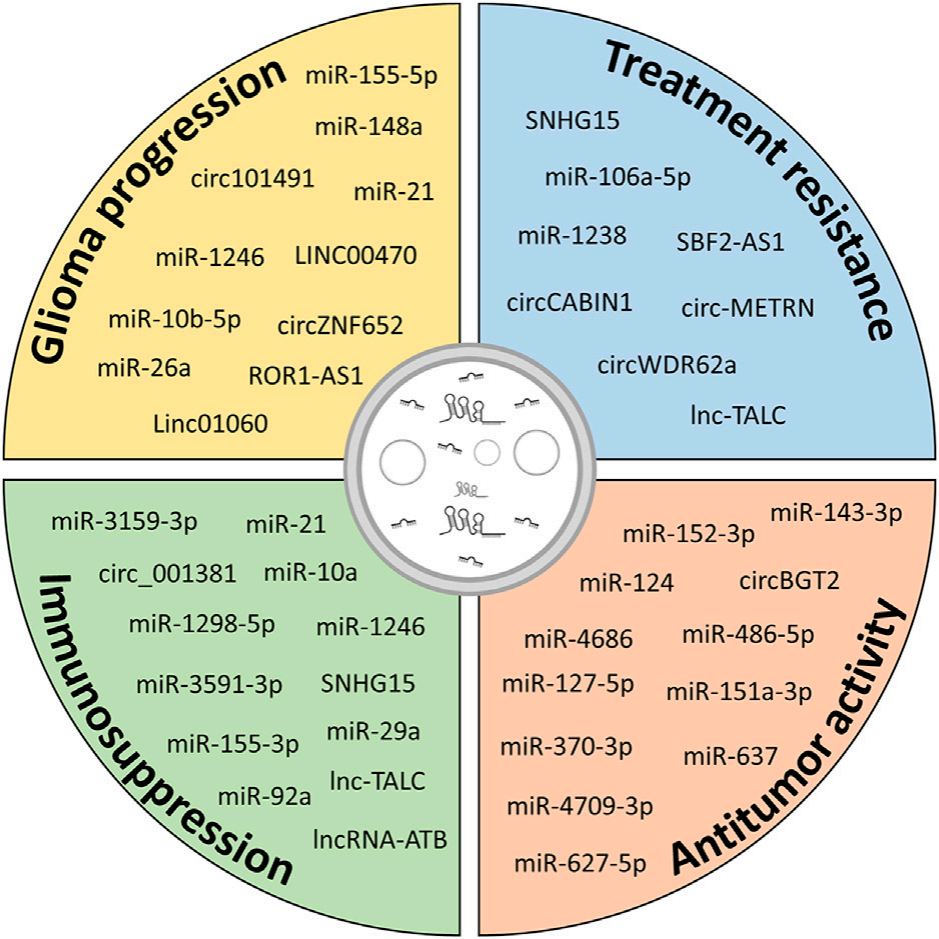
Potential contribution of ncRNAs in different aspects of glioma biology. Exosomal ncRNAs released from glioma cells and cells of the TME contribute to glioma progression by increasing growth, migration and invasiveness, inducing resistance to therapy and immunosuppressive functions in cells of the TME. However, modulation of specific exosomal ncRNAs or administration of exogenous ncRNAs can be exploited to hinder glioma progression.

Altogether, these data (summarized in Table 2) indicate that EV-mediated interactions between cells of the TME and glioma influence various aspects of tumor behavior, including migration, invasion, proliferation, autophagy, vitality, and immune modulation, through the transfer of ncRNAs. These EV-mediated communications can be exploited for developing targeted interventions on specific ncRNAs aimed at disrupting this complex crosstalk and potentially halting glioma progression (Figure 4).

**TABLE 2:** Summary of TME-derived exosomal ncRNAs, their targets and effects on glioma cells.

Source	Recipient cells	ncRNAs	ncRNA targets	Mechanism of action	References
Rat primary astrocytes	rat glioma C6 cells	miR-152-3p and miR-143-3p	Dnmt1, Mmp3, Runx2, Hk2, Nras, Bag3	Reduction of glioma cell migration and invasion	Fukuda et al. (2021)
Mouse primary astrocytes	murine glioma GL261 cells	miR-124	VRAC	Reduction of glioma cell viability	Serpe et al. (2021)
Astrocytes (TME)	Brain metastasis	miR-17–92 cluster	PTEN	Recruitment of myeloid cells with pro-metastasis properties	Zhang et al. (2015)
RBP-J overexpressing THP-1 macrophages	U373 and U87 glioma cell lines	circBTG2	miR-25-3p/PTEN	Suppresion of suppress glioma cell proliferation and invasion	Shi et al. (2022)
Astrocytes (TME)	Brain metastasis	miR-17–92 cluster	PTEN	Recruitment of myeloid cto ells with pro-metastasis properties	Zhang et al. (2015)
BV2 microglial cell line	U373 and U87 glioma cells and mouse astrocytes	miR-124	Unknown	Reduction of tumor cell migration and astrogliosis and recruitment of NK cell	Hong et al. (2021)
BV2 microglial cell line	murine glioma GL261 cells	miR-7239-3p	Bmal1	Promotion of glioma growth, migration, and invasion	Li et al. (2021b)
Human THP1 macrophage cell line	T98 and U251-MG cell lines	miR-15a and miR-92a	Ccnd1 and Rap1b	Blocking mTOR signaling pathway to inhibit glioma invasion and migration	Yao et al. (2021)
GA-hMSCs	Human glioma stem cells	miR-1587	Ncor1	Increase in tumor volume and reduced survival	Figueroa et al. (2017)

## 5 Conclusion

In this review, we have highlighted the role of ncRNAs in the pathogenesis of glioma. In cancer, miRNAs have been described as either oncomiR or tumor suppressors, and in several cases, they have shown both effects; this paradoxical behavior is due to their ability to bind and inhibit hundreds of different target transcripts. The finding that miRNAs can be released in EVs, reach other cells, and modulate their gene expression widens their role in tumor progression, invasiveness, immune escape, angiogenesis, and resistance to drugs. We have described that, while glioma uses this communication route to make the TME more compliant to its survival and development, immune cells may use the same weapon to counteract glioma spreading; the unbalance between these forces determines the clinical outcome. LncRNAs or circRNAs, analogously stored in EVs, can modulate miRNAs functions in a paracrine fashion, acting as molecular sponges. This underlines the importance of post-transcriptional regulators as complex miRNA-lncRNA-transcript multidirectional networks, in which both the well-known immune cells of the TME, and progenitor cells, such as OPCs, play important roles. Immunotherapy is a clear example of treatment that targets the TME instead of cancer cells (Murciano-Goroff et al., 2020). The increasing advances in gene therapy and RNA technologies are likely to further revolutionize the current therapeutic approach to glioma. We have reported several studies in which ncRNAs have been used as pharmacological agents and have been shown to rescue specific mechanisms of glioma progression also acting on the TME. To translate these findings into RNA-based therapeutics, several challenges still have to be addressed, starting from their delivery into the glioma, considering that brain is not an easily accessible organ. In this context, tropism of NPCs and their exosomes for hypoxic tumor areas provides a new opportunity for glioma therapy. In addition to nano-drug delivery platforms that use polymers or liposome-based formulations, both natural and engineered EVs have been developed and they are increasingly improving, with better biocompatibility, prolonged blood circulation life span, lower immunogenicity and systemic toxicity (Liu and Su, 2019; Cabeza et al., 2020). Cultured cells can be genetically modified to produce exosomes that express surface proteins with increased BBB permeability or enhanced glioma tropism (Basu and Ghosh, 2019). Recently, engineered exosome producer cells implanted in a mouse model of Parkinson disease successfully prevented 6-OHDA-induced cell death by efficient production and delivery of exosomes containing catalase mRNA into the brain, opening the door for new ways to achieve therapeutic effects.

A number of therapeutic approaches utilizing EVs have already been tested for the treatment of diseases including solid tumors and neurodegenerative disorders (Rezaie et al., 2022). In the near future, we expect that mimicking or counteracting EV-based communication will become a concrete therapeutic means for glioma patients.

## References

[B1] Abels E. R. Maas S. L. N. Nieland L. Wei Z. Cheah P. S. Tai E. (2019). Glioblastoma-associated microglia reprogramming is mediated by functional transfer of extracellular miR-21. Cell Rep. 28, 3105–3119. 10.1016/j.celrep.2019.08.036 31533034 PMC6817978

[B2] Adamus T. Hung C. Y. Yu C. Kang E. Hammad M. Flores L. (2022). Glioma-targeted delivery of exosome-encapsulated antisense oligonucleotides using neural stem cells. Mol. Ther. Nucleic Acids 27, 611–620. 10.1016/j.omtn.2021.12.029 35036069 PMC8752899

[B3] Angelini J. Marangon D. Raffaele S. Lecca D. Abbracchio M. P. (2021). The distribution of GPR17-expressing cells correlates with white matter inflammation status in brain tissues of multiple sclerosis patients. Int. J. Mol. Sci. 22, 4574. 10.3390/ijms22094574 33925469 PMC8123849

[B4] Bao Z. Zhang N. Niu W. Mu M. Zhang X. Hu S. (2022). Exosomal miR-155-5p derived from glioma stem-like cells promotes mesenchymal transition via targeting ACOT12. Cell Death Dis. 13, 725. 10.1038/s41419-022-05097-w 35986010 PMC9391432

[B5] Basu B. Ghosh M. K. (2019). Extracellular vesicles in glioma: from diagnosis to therapy. Bioessays 41, e1800245. 10.1002/bies.201800245 31188499

[B6] Bian E. B. Chen E. F. Xu Y. D. Yang Z. H. Tang F. Ma C. C. (2019). Exosomal lncRNA-ATB activates astrocytes that promote glioma cell invasion. Int. J. Oncol. 54, 713–721. 10.3892/ijo.2018.4644 30483768

[B7] Brooks L. J. Clements M. P. Burden J. J. Kocher D. Richards L. Devesa S. C. (2021). The white matter is a pro-differentiative niche for glioblastoma. Nat. Commun. 12, 2184. 10.1038/s41467-021-22225-w 33846316 PMC8042097

[B8] Buruiana A. Florian S. I. Florian A. I. Timis T. L. Mihu C. M. Miclaus M. (2020). The roles of miRNA in glioblastoma tumor cell communication: diplomatic and aggressive negotiations. Int. J. Mol. Sci. 21, 1950. 10.3390/ijms21061950 32178454 PMC7139390

[B9] Cabeza L. Perazzoli G. Pena M. Cepero A. Luque C. Melguizo C. (2020). Cancer therapy based on extracellular vesicles as drug delivery vehicles. J. Control Release 327, 296–315. 10.1016/j.jconrel.2020.08.018 32814093

[B10] Cai Q. Zhu A. Gong L. (2018). Exosomes of glioma cells deliver miR-148a to promote proliferation and metastasis of glioblastoma via targeting CADM1. Bull. Cancer 105, 643–651. 10.1016/j.bulcan.2018.05.003 29921422

[B11] Caruso F. P. Garofano L. D'angelo F. Yu K. Tang F. Yuan J. (2020). A map of tumor-host interactions in glioma at single-cell resolution. Gigascience 9, giaa109. 10.1093/gigascience/giaa109 33155039 PMC7645027

[B12] Catalano M. Serpe C. Limatola C. (2022). Microglial extracellular vesicles as modulators of brain microenvironment in glioma. Int. J. Mol. Sci. 23, 13165. 10.3390/ijms232113165 36361947 PMC9656645

[B13] Chai Y. Wu H. T. Liang C. D. You C. Y. Xie M. X. Xiao S. W. (2020). Exosomal lncRNA ROR1-AS1 derived from tumor cells promotes glioma progression via regulating miR-4686. Int. J. Nanomedicine 15, 8863–8872. 10.2147/IJN.S271795 33204092 PMC7667171

[B14] Cheng J. Meng J. Zhu L. Peng Y. (2020). Exosomal noncoding RNAs in Glioma: biological functions and potential clinical applications. Mol. Cancer 19, 66. 10.1186/s12943-020-01189-3 32213181 PMC7098115

[B15] Chulpanova D. S. Pukhalskaia T. V. Rizvanov A. A. Solovyeva V. V. (2022). Contribution of tumor-derived extracellular vesicles to malignant transformation of normal cells. Bioeng. (Basel) 9, 245. 10.3390/bioengineering9060245 PMC9220176 35735488

[B16] Domenis R. Cesselli D. Toffoletto B. Bourkoula E. Caponnetto F. Manini I. (2017). Systemic T cells immunosuppression of glioma stem cell-derived exosomes is mediated by monocytic myeloid-derived suppressor cells. PLoS One 12, e0169932. 10.1371/journal.pone.0169932 28107450 PMC5249124

[B17] Fabbiano F. Corsi J. Gurrieri E. Trevisan C. Notarangelo M. D'agostino V. G. (2020). RNA packaging into extracellular vesicles: an orchestra of RNA-binding proteins? J. Extracell. Vesicles 10, e12043. 10.1002/jev2.12043 33391635 PMC7769857

[B18] Feng Y. Ye Z. Song F. He Y. Liu J. (2022). The role of TAMs in tumor microenvironment and new research progress. Stem Cells Int. 2022, 5775696. 10.1155/2022/5775696 36004381 PMC9395242

[B19] Figueroa J. Phillips L. M. Shahar T. Hossain A. Gumin J. Kim H. (2017). Exosomes from glioma-associated mesenchymal stem cells increase the tumorigenicity of glioma stem-like cells via transfer of miR-1587. Cancer Res. 77, 5808–5819. 10.1158/0008-5472.CAN-16-2524 28855213 PMC5668150

[B20] Fukuda S. Akiyama M. Niki Y. Kawatsura R. Harada H. Nakahama K. I. (2021). Inhibitory effects of miRNAs in astrocytes on C6 glioma progression via connexin 43. Mol. Cell Biochem. 476, 2623–2632. 10.1007/s11010-021-04118-0 33660186

[B21] Gabrielli M. Raffaele S. Fumagalli M. Verderio C. (2022). The multiple faces of extracellular vesicles released by microglia: where are we 10 years after? Front. Cell Neurosci. 16, 984690. 10.3389/fncel.2022.984690 36176630 PMC9514840

[B22] Geng X. Zhang Y. Lin X. Zeng Z. Hu J. Hao L. (2022). Exosomal circWDR62 promotes temozolomide resistance and malignant progression through regulation of the miR-370-3p/MGMT axis in glioma. Cell Death Dis. 13, 596. 10.1038/s41419-022-05056-5 35817771 PMC9273787

[B23] Glass R. Synowitz M. Kronenberg G. Walzlein J. H. Markovic D. S. Wang L. P. (2005). Glioblastoma-induced attraction of endogenous neural precursor cells is associated with improved survival. J. Neurosci. 25, 2637–2646. 10.1523/JNEUROSCI.5118-04.2005 15758174 PMC6725181

[B24] Guo X. Qiu W. Liu Q. Qian M. Wang S. Zhang Z. (2018). Immunosuppressive effects of hypoxia-induced glioma exosomes through myeloid-derived suppressor cells via the miR-10a/Rora and miR-21/Pten Pathways. Oncogene 37, 4239–4259. 10.1038/s41388-018-0261-9 29713056

[B25] Guo X. Qiu W. Wang J. Liu Q. Qian M. Wang S. (2019). Glioma exosomes mediate the expansion and function of myeloid-derived suppressor cells through microRNA-29a/Hbp1 and microRNA-92a/Prkar1a pathways. Int. J. Cancer 144, 3111–3126. 10.1002/ijc.32052 30536597

[B26] Gurung S. Perocheau D. Touramanidou L. Baruteau J. (2021). The exosome journey: from biogenesis to uptake and intracellular signalling. Cell Commun. Signal 19, 47. 10.1186/s12964-021-00730-1 33892745 PMC8063428

[B27] Han Y. Liu Y. Zhang B. Yin G. (2021). Exosomal circRNA 0001445 promotes glioma progression through miRNA-127-5p/SNX5 pathway. Aging (Albany NY) 13, 13287–13299. 10.18632/aging.203013 33982667 PMC8148472

[B28] Henrik Heiland D. Ravi V. M. Behringer S. P. Frenking J. H. Wurm J. Joseph K. (2019). Tumor-associated reactive astrocytes aid the evolution of immunosuppressive environment in glioblastoma. Nat. Commun. 10, 2541. 10.1038/s41467-019-10493-6 31186414 PMC6559986

[B29] Hide T. Komohara Y. Miyasato Y. Nakamura H. Makino K. Takeya M. (2018). Oligodendrocyte progenitor cells and macrophages/microglia produce glioma stem cell niches at the tumor border. EBioMedicine 30, 94–104. 10.1016/j.ebiom.2018.02.024 29559295 PMC5952226

[B30] Hong S. You J. Y. Paek K. Park J. Kang S. J. Han E. H. (2021). Inhibition of tumor progression and M2 microglial polarization by extracellular vesicle-mediated microRNA-124 in a 3D microfluidic glioblastoma microenvironment. Theranostics 11, 9687–9704. 10.7150/thno.60851 34646393 PMC8490520

[B31] Huo L. Du X. Li X. Liu S. Xu Y. (2021). The emerging role of neural cell-derived exosomes in intercellular communication in health and neurodegenerative diseases. Front. Neurosci. 15, 738442. 10.3389/fnins.2021.738442 34531720 PMC8438217

[B32] Jeppesen D. K. Zhang Q. Franklin J. L. Coffey R. J. (2023). Extracellular vesicles and nanoparticles: emerging complexities. Trends Cell Biol. 33, 667–681. 10.1016/j.tcb.2023.01.002 36737375 PMC10363204

[B33] John Lin C. C. Yu K. Hatcher A. Huang T. W. Lee H. K. Carlson J. (2017). Identification of diverse astrocyte populations and their malignant analogs. Nat. Neurosci. 20, 396–405. 10.1038/nn.4493 28166219 PMC5824716

[B34] Kosaka N. Iguchi H. Hagiwara K. Yoshioka Y. Takeshita F. Ochiya T. (2013). Neutral sphingomyelinase 2 (nSMase2)-dependent exosomal transfer of angiogenic microRNAs regulate cancer cell metastasis. J. Biol. Chem. 288, 10849–10859. 10.1074/jbc.M112.446831 23439645 PMC3624465

[B35] Lang H. L. Hu G. W. Zhang B. Kuang W. Chen Y. Wu L. (2017). Glioma cells enhance angiogenesis and inhibit endothelial cell apoptosis through the release of exosomes that contain long non-coding RNA CCAT2. Oncol. Rep. 38, 785–798. 10.3892/or.2017.5742 28656228 PMC5562059

[B36] Lecca D. Marangon D. Coppolino G. T. Mendez A. M. Finardi A. Costa G. D. (2016). MiR-125a-3p timely inhibits oligodendroglial maturation and is pathologically up-regulated in human multiple sclerosis. Sci. Rep. 6, 34503. 10.1038/srep34503 27698367 PMC5048305

[B37] Li J. Liao T. Liu H. Yuan H. Ouyang T. Wang J. (2021a). Hypoxic glioma stem cell-derived exosomes containing Linc01060 promote progression of glioma by regulating the MZF1/c-myc/HIF1α Axis. Cancer Res. 81, 114–128. 10.1158/0008-5472.CAN-20-2270 33158815

[B38] Li J. Yuan H. Xu H. Zhao H. Xiong N. (2020). Hypoxic cancer-secreted exosomal miR-182-5p promotes glioblastoma angiogenesis by targeting kruppel-like factor 2 and 4. Mol. Cancer Res. 18, 1218–1231. 10.1158/1541-7786.MCR-19-0725 32366676

[B39] Li M. Xu H. Qi Y. Pan Z. Li B. Gao Z. (2022). Tumor-derived exosomes deliver the tumor suppressor miR-3591-3p to induce M2 macrophage polarization and promote glioma progression. Oncogene 41, 4618–4632. 10.1038/s41388-022-02457-w 36085418 PMC9546774

[B40] Li X. Guan J. Jiang Z. Cheng S. Hou W. Yao J. (2021b). Microglial exosome miR-7239-3p promotes glioma progression by regulating circadian genes. Neurosci. Bull. 37, 497–510. 10.1007/s12264-020-00626-z 33528793 PMC8055789

[B41] Li Y. Li W. Zeng X. Tang X. Zhang S. Zhong F. (2019a). The role of microRNA-148a and downstream DLGAP1 on the molecular regulation and tumor progression on human glioblastoma. Oncogene 38, 7234–7248. 10.1038/s41388-019-0922-3 31477833

[B42] Li Z. Meng X. Wu P. Zha C. Han B. Li L. (2021c). Glioblastoma cell-derived lncRNA-containing exosomes induce microglia to produce complement C5, promoting chemotherapy resistance. Cancer Immunol. Res. 9, 1383–1399. 10.1158/2326-6066.CIR-21-0258 34667108

[B43] Li Z. Zhang J. Zheng H. Li C. Xiong J. Wang W. (2019b). Modulating lncRNA SNHG15/CDK6/miR-627 circuit by palbociclib, overcomes temozolomide resistance and reduces M2-polarization of glioma associated microglia in glioblastoma multiforme. J. Exp. Clin. Cancer Res. 38, 380. 10.1186/s13046-019-1371-0 31462285 PMC6714301

[B44] Liu C. Su C. (2019). Design strategies and application progress of therapeutic exosomes. Theranostics 9, 1015–1028. 10.7150/thno.30853 30867813 PMC6401399

[B45] Liu L. Xiao S. Wang Y. Zhu Z. Cao Y. Yang S. (2022). Identification of a novel circular RNA circZNF652/miR-486-5p/SERPINE1 signaling cascade that regulates cancer aggressiveness in glioblastoma (GBM). Bioengineered 13, 1411–1423. 10.1080/21655979.2021.2018096 35258403 PMC8805984

[B46] Liu S. Q. Jiang S. Li C. Zhang B. Li Q. J. (2014). miR-17-92 cluster targets phosphatase and tensin homology and Ikaros Family Zinc Finger 4 to promote TH17-mediated inflammation. J. Biol. Chem. 289, 12446–12456. 10.1074/jbc.M114.550723 24644282 PMC4007439

[B47] Liu X. Guo Q. Gao G. Cao Z. Guan Z. Jia B. (2023). Exosome-transmitted circCABIN1 promotes temozolomide resistance in glioblastoma via sustaining ErbB downstream signaling. J. Nanobiotechnology 21, 45. 10.1186/s12951-023-01801-w 36755314 PMC9906870

[B48] Liu Y. J. Wang C. (2023). A review of the regulatory mechanisms of extracellular vesicles-mediated intercellular communication. Cell Commun. Signal 21, 77. 10.1186/s12964-023-01103-6 37055761 PMC10100201

[B49] Ma B. Wang S. Wu W. Shan P. Chen Y. Meng J. (2023). Mechanisms of circRNA/lncRNA-miRNA interactions and applications in disease and drug research. Biomed. Pharmacother. 162, 114672. 10.1016/j.biopha.2023.114672 37060662

[B50] Ma C. Nguyen H. P. T. Jones J. J. Stylli S. S. Whitehead C. A. Paradiso L. (2022). Extracellular vesicles secreted by glioma stem cells are involved in radiation resistance and glioma progression. Int. J. Mol. Sci. 23, 2770. 10.3390/ijms23052770 35269915 PMC8911495

[B51] Ma W. Zhou Y. Liu M. Qin Q. Cui Y. (2021). Long non-coding RNA LINC00470 in serum derived exosome: a critical regulator for proliferation and autophagy in glioma cells. Cancer Cell Int. 21, 149. 10.1186/s12935-021-01825-y 33663509 PMC7931344

[B52] Marangon D. Abbracchio M. P. Lecca D. (2021). Pathway-focused profiling of oligodendrocytes over-expressing miR-125a-3p reveals alteration of wnt and cell-to-cell signaling. Cell Mol. Neurobiol. 41, 105–114. 10.1007/s10571-020-00836-z 32239390 PMC11448621

[B53] Marangon D. Castro E Silva J. H. Lecca D. (2022). Neuronal and glial communication via non-coding RNAs: messages in extracellular vesicles. Int. J. Mol. Sci. 24, 470. 10.3390/ijms24010470 36613914 PMC9820657

[B54] Mega A. Hartmark Nilsen M. Leiss L. W. Tobin N. P. Miletic H. Sleire L. (2020). Astrocytes enhance glioblastoma growth. Glia 68, 316–327. 10.1002/glia.23718 31509308

[B55] Messenger S. W. Woo S. S. Sun Z. Martin T. F. J. (2018). A Ca(2+)-stimulated exosome release pathway in cancer cells is regulated by Munc13-4. J. Cell Biol. 217, 2877–2890. 10.1083/jcb.201710132 29930202 PMC6080937

[B56] Misir S. Wu N. Yang B. B. (2022). Specific expression and functions of circular RNAs. Cell Death Differ. 29, 481–491. 10.1038/s41418-022-00948-7 35169296 PMC8901656

[B57] Muller L. Mitsuhashi M. Simms P. Gooding W. E. Whiteside T. L. (2016). Tumor-derived exosomes regulate expression of immune function-related genes in human T cell subsets. Sci. Rep. 6, 20254. 10.1038/srep20254 26842680 PMC4740743

[B58] Murciano-Goroff Y. R. Warner A. B. Wolchok J. D. (2020). The future of cancer immunotherapy: microenvironment-targeting combinations. Cell Res. 30, 507–519. 10.1038/s41422-020-0337-2 32467593 PMC7264181

[B93] Nix J. S. Yuan M. Imada E. L. Ames H. Marchionni L. Gutmann D. H. (2021). Global microRNA profiling identified miR-10b-5p as a regulator of neurofibromatosis 1 (NF1)-glioma migration. Neuropathology and Applied Neurobiology 47 (1), 96–107. 10.1111/nan.12641 32603552

[B59] O'brien K. Breyne K. Ughetto S. Laurent L. C. Breakefield X. O. (2020). RNA delivery by extracellular vesicles in mammalian cells and its applications. Nat. Rev. Mol. Cell Biol. 21, 585–606. 10.1038/s41580-020-0251-y 32457507 PMC7249041

[B60] Ong D. S. T. Hu B. Ho Y. W. Sauve C. G. Bristow C. A. Wang Q. (2017). PAF promotes stemness and radioresistance of glioma stem cells. Proc. Natl. Acad. Sci. U. S. A. 114, E9086–E9095. 10.1073/pnas.1708122114 29073105 PMC5664518

[B61] Parmigiani E. Scalera M. Mori E. Tantillo E. Vannini E. (2021). Old stars and new players in the brain tumor microenvironment. Front. Cell Neurosci. 15, 709917. 10.3389/fncel.2021.709917 34690699 PMC8527006

[B62] Qi Y. Jin C. Qiu W. Zhao R. Wang S. Li B. (2022). The dual role of glioma exosomal microRNAs: glioma eliminates tumor suppressor miR-1298-5p via exosomes to promote immunosuppressive effects of MDSCs. Cell Death Dis. 13, 426. 10.1038/s41419-022-04872-z 35501306 PMC9061735

[B63] Qian C. Wang Y. Ji Y. Chen D. Wang C. Zhang G. (2022). Neural stem cell-derived exosomes transfer miR-124-3p into cells to inhibit glioma growth by targeting FLOT2. Int. J. Oncol. 61, 115. 10.3892/ijo.2022.5405 35929514 PMC9387557

[B64] Qiu W. Guo X. Li B. Wang J. Qi Y. Chen Z. (2021). Exosomal miR-1246 from glioma patient body fluids drives the differentiation and activation of myeloid-derived suppressor cells. Mol. Ther. 29, 3449–3464. 10.1016/j.ymthe.2021.06.023 34217892 PMC8636176

[B65] Rajabi A. Kayedi M. Rahimi S. Dashti F. Mirazimi S. M. A. Homayoonfal M. (2022). Non-coding RNAs and glioma: focus on cancer stem cells. Mol. Ther. Oncolytics 27, 100–123. 10.1016/j.omto.2022.09.005 36321132 PMC9593299

[B66] Rezaie J. Feghhi M. Etemadi T. (2022). A review on exosomes application in clinical trials: perspective, questions, and challenges. Cell Commun. Signal 20, 145. 10.1186/s12964-022-00959-4 36123730 PMC9483361

[B67] Serpe C. Michelucci A. Monaco L. Rinaldi A. De Luca M. Familiari P. (2022). Astrocytes-derived small extracellular vesicles hinder glioma growth. Biomedicines 10, 2952. 10.3390/biomedicines10112952 36428520 PMC9688032

[B68] Serpe C. Monaco L. Relucenti M. Iovino L. Familiari P. Scavizzi F. (2021). Microglia-derived small extracellular vesicles reduce glioma growth by modifying tumor cell metabolism and enhancing glutamate clearance through miR-124. Cells 10, 2066. 10.3390/cells10082066 34440835 PMC8393731

[B69] Sharma P. Aaroe A. Liang J. Puduvalli V. K. (2023). Tumor microenvironment in glioblastoma: current and emerging concepts. Neurooncol Adv. 5, vdad009. 10.1093/noajnl/vdad009 36968288 PMC10034917

[B70] Shi L. Cao Y. Yuan W. Guo J. Sun G. (2022). Exosomal circRNA BTG2 derived from RBP-J overexpressed-macrophages inhibits glioma progression via miR-25-3p/PTEN. Cell Death Dis. 13, 506. 10.1038/s41419-022-04908-4 35643814 PMC9148311

[B71] Statello L. Guo C. J. Chen L. L. Huarte M. (2021). Gene regulation by long non-coding RNAs and its biological functions. Nat. Rev. Mol. Cell Biol. 22, 96–118. 10.1038/s41580-020-00315-9 33353982 PMC7754182

[B72] Stupp R. Van Den Bent M. J. Hegi M. E. (2005). Optimal role of temozolomide in the treatment of malignant gliomas. Curr. Neurol. Neurosci. Rep. 5, 198–206. 10.1007/s11910-005-0047-7 15865885

[B73] Sun X. Ma X. Wang J. Zhao Y. Wang Y. Bihl J. C. (2017). Glioma stem cells-derived exosomes promote the angiogenic ability of endothelial cells through miR-21/VEGF signal. Oncotarget 8, 36137–36148. 10.18632/oncotarget.16661 28410224 PMC5482644

[B74] Taylor O. G. Brzozowski J. S. Skelding K. A. (2019). Glioblastoma multiforme: an overview of emerging therapeutic targets. Front. Oncol. 9, 963. 10.3389/fonc.2019.00963 31616641 PMC6775189

[B75] Van Der Vos K. E. Abels E. R. Zhang X. Lai C. Carrizosa E. Oakley D. (2016). Directly visualized glioblastoma-derived extracellular vesicles transfer RNA to microglia/macrophages in the brain. Neuro Oncol. 18, 58–69. 10.1093/neuonc/nov244 26433199 PMC4677420

[B76] Wang G. Zhong K. Wang Z. Zhang Z. Tang X. Tong A. (2022). Tumor-associated microglia and macrophages in glioblastoma: from basic insights to therapeutic opportunities. Front. Immunol. 13, 964898. 10.3389/fimmu.2022.964898 35967394 PMC9363573

[B77] Wang H. Zhou H. Xu J. Lu Y. Ji X. Yao Y. (2021a). Different T-cell subsets in glioblastoma multiforme and targeted immunotherapy. Cancer Lett. 496, 134–143. 10.1016/j.canlet.2020.09.028 33022290

[B78] Wang M. Zhao Y. Yu Z. Y. Zhang R. D. Li S. A. Zhang P. (2020). Glioma exosomal microRNA-148a-3p promotes tumor angiogenesis through activating the EGFR/MAPK signaling pathway via inhibiting ERRFI1. Cancer Cell Int. 20, 518. 10.1186/s12935-020-01566-4 33117083 PMC7590612

[B79] Wang X. Cao Q. Shi Y. Wu X. Mi Y. Liu K. (2021b). Identification of low-dose radiation-induced exosomal circ-METRN and miR-4709-3p/GRB14/PDGFRα pathway as a key regulatory mechanism in Glioblastoma progression and radioresistance: functional validation and clinical theranostic significance. Int. J. Biol. Sci. 17, 1061–1078. 10.7150/ijbs.57168 33867829 PMC8040305

[B80] Wang Z. F. Liao F. Wu H. Dai J. (2019). Glioma stem cells-derived exosomal miR-26a promotes angiogenesis of microvessel endothelial cells in glioma. J. Exp. Clin. Cancer Res. 38, 201. 10.1186/s13046-019-1181-4 31101062 PMC6525364

[B81] Wu C. Su J. Long W. Qin C. Wang X. Xiao K. (2020). LINC00470 promotes tumour proliferation and invasion, and attenuates chemosensitivity through the LINC00470/miR-134/Myc/ABCC1 axis in glioma. J. Cell Mol. Med. 24, 12094–12106. 10.1111/jcmm.15846 32916774 PMC7579701

[B82] Wu P. Guo J. Yang H. Yuan D. Wang C. Wang Z. (2022). Exosomes derived from hypoxic glioma cells reduce the sensitivity of glioma cells to temozolomide through carrying miR-106a-5p. Drug Des. Devel Ther. 16, 3589–3598. 10.2147/DDDT.S382690 PMC9556335 36248244

[B83] Xu H. Li M. Pan Z. Zhang Z. Gao Z. Zhao R. (2022). miR-3184-3p enriched in cerebrospinal fluid exosomes contributes to progression of glioma and promotes M2-like macrophage polarization. Cancer Sci. 113, 2668–2680. 10.1111/cas.15372 35411604 PMC9357622

[B84] Xu J. Zhang J. Zhang Z. Gao Z. Qi Y. Qiu W. (2021). Hypoxic glioma-derived exosomes promote M2-like macrophage polarization by enhancing autophagy induction. Cell Death Dis. 12, 373. 10.1038/s41419-021-03664-1 33828078 PMC8026615

[B85] Yang C. H. Yue J. Pfeffer S. R. Fan M. Paulus E. Hosni-Ahmed A. (2014). MicroRNA-21 promotes glioblastoma tumorigenesis by down-regulating insulin-like growth factor-binding protein-3 (IGFBP3). J. Biol. Chem. 289, 25079–25087. 10.1074/jbc.M114.593863 25059666 PMC4155674

[B86] Yao J. Wang Z. Cheng Y. Ma C. Zhong Y. Xiao Y. (2021). M2 macrophage-derived exosomal microRNAs inhibit cell migration and invasion in gliomas through PI3K/AKT/mTOR signaling pathway. J. Transl. Med. 19, 99. 10.1186/s12967-021-02766-w 33676540 PMC7937290

[B87] Yin J. Zeng A. Zhang Z. Shi Z. Yan W. You Y. (2019). Exosomal transfer of miR-1238 contributes to temozolomide-resistance in glioblastoma. EBioMedicine 42, 238–251. 10.1016/j.ebiom.2019.03.016 30917935 PMC6491393

[B88] Zhang C. Zhou Y. Gao Y. Zhu Z. Zeng X. Liang W. (2022). Radiated glioblastoma cell-derived exosomal circ_0012381 induce M2 polarization of microglia to promote the growth of glioblastoma by CCL2/CCR2 axis. J. Transl. Med. 20, 388. 10.1186/s12967-022-03607-0 36058942 PMC9441045

[B89] Zhang L. Zhang S. Yao J. Lowery F. J. Zhang Q. Huang W. C. (2015). Microenvironment-induced PTEN loss by exosomal microRNA primes brain metastasis outgrowth. Nature 527, 100–104. 10.1038/nature15376 26479035 PMC4819404

[B90] Zhang P. Xia Q. Liu L. Li S. Dong L. (2020). Current opinion on molecular characterization for GBM classification in guiding clinical diagnosis, prognosis, and therapy. Front. Mol. Biosci. 7, 562798. 10.3389/fmolb.2020.562798 33102518 PMC7506064

[B91] Zhang X. H. Song Y. C. Qiu F. Wang Z. C. Li N. Zhao F. B. (2023). Hypoxic glioma cell-secreted exosomal circ101491 promotes the progression of glioma by regulating miR-125b-5p/EDN1. Brain Res. Bull. 195, 55–65. 10.1016/j.brainresbull.2023.02.006 36796652

[B92] Zhang Z. Yin J. Lu C. Wei Y. Zeng A. You Y. (2019). Exosomal transfer of long non-coding RNA SBF2-AS1 enhances chemoresistance to temozolomide in glioblastoma. J. Exp. Clin. Cancer Res. 38, 166. 10.1186/s13046-019-1139-6 30992025 PMC6469146

